# An mHealth App (Speech Banana) for Auditory Training: App Design and Development Study

**DOI:** 10.2196/20890

**Published:** 2021-03-15

**Authors:** J Tilak Ratnanather, Rohit Bhattacharya, Margo B Heston, Joanne Song, Lindsey R Fernandez, Hong Seo Lim, Seung-Wook Lee, Edric Tam, Sungho Yoo, Seung-Ho Bae, Inez Lam, Hyoung Won Jeon, Son A Chang, Ja-Won Koo

**Affiliations:** 1 Center for Imaging Science and Institute for Computational Medicine Department of Biomedical Engineering Johns Hopkins University Baltimore, MD United States; 2 Department of Otorhinolaryngology Seoul National University College of Medicine Seoul National University Bundang Hospital Seongnam-si Republic of Korea; 3 Soree Ear Clinic Rehabilitation Center Seoul Republic of Korea

**Keywords:** speech therapy, mobile phone, computers, handheld, cochlear implants, hearing aids

## Abstract

**Background:**

With the growing adult population using electronic hearing devices such as cochlear implants or hearing aids, there is an increasing worldwide need for auditory training (AT) to promote optimal device use. However, financial resources and scheduling conflicts make clinical AT infeasible.

**Objective:**

To address this gap between need and accessibility, we primarily aimed to develop a mobile health (mHealth) app called Speech Banana for AT. The app would be substantially more affordable and portable than clinical AT; would deliver a validated training model that is reflective of modern techniques; and would track users’ progress in speech comprehension, providing greater continuity between periodic in-person visits. To improve international availability, our secondary aim was to implement the English language training model into Korean as a proof of concept for worldwide usability.

**Methods:**

A problem- and objective-centered Design Science Research Methodology approach was adopted to develop the Speech Banana app. A review of previous literature and computer-based learning programs outlined current AT gaps, whereas interviews with speech pathologists and users clarified the features that were addressed in the app. Past and present users were invited to evaluate the app via community forums and the System Usability Scale.

**Results:**

Speech Banana has been implemented in English and Korean languages for iPad and web use. The app comprises 38 lessons, which include analytic exercises pairing visual and auditory stimuli, and synthetic quizzes presenting auditory stimuli only. During quizzes, users type the sentence heard, and the app provides visual feedback on performance. Users may select a male or female speaker and the volume of background noise, allowing for training with a range of frequencies and signal-to-noise ratios. There were more than 3200 downloads of the English iPad app and almost 100 downloads of the Korean app; more than 100 users registered for the web apps. The English app received a System Usability Scale rating of “good” from 6 users, and the Korean app received a rating of “OK” from 16 users.

**Conclusions:**

Speech Banana offers AT accessibility with a validated curriculum, allowing users to develop speech comprehension skills with the aid of a mobile device. This mHealth app holds potential as a supplement to clinical AT, particularly in this era of global telemedicine.

## Introduction

### Background

Although auditory prostheses such as hearing aids and cochlear implants have become widely available, several challenges impede adult users from attaining effective hearing rehabilitation. These challenges stem from speech comprehension difficulties, leading to inconsistent device use [1]. Evidence suggests that clinical auditory training (AT) augmented with software programs may improve speech comprehension; however, AT remains globally inaccessible because of financial and clinical time constraints [2-8]. For instance, in the United States, public insurance programs such as Medicare and Medicaid provide insufficient funding to cover the cost of AT [9]. In Korea, children up to 19 years of age may receive AT, but adults are not covered by national health insurance [10]. In the United Kingdom, a lack of AT coverage can leave adult cochlear implant recipients without access to regular sessions outside of annual or semiannual mapping appointments [11,12]. Although clinics attempt to absorb costs through fundraising, sessions may still be prohibitively expensive, leading adults to forgo these services [9]. Even if a patient can afford AT, weekly sessions may require adults to take time away from work, thereby reducing productivity and further exacerbating financial stress [13]. As a result, adults worldwide use their devices without the guidance of a clinician. This motivates the need for accessible AT and for multilingual availability.

One way to address these concerns is to digitize AT and automate the recorded progress. Several groups have developed desktop AT apps, many of which contain proprietary software with management control by clinics [14-16]. All apps use a combination of synthetic (bottom-up) and analytical (top-down) approaches with varying complexity levels; however, they focus primarily on words and phrases, which cultivate skills relevant to the app but not necessarily to daily life. Although desktop-based AT apps are in use, fewer apps are built for mobile devices that are ubiquitous worldwide, such as phones and tablets [17-20]. One free desktop app called i-AngelSound has been redesigned for iPad (Apple Inc) but is not available on other platforms [21,22]. Additional tablet-based apps have emerged for rehabilitating aphasia patients [23,24] and audiological management [25], which indicates a clinical interest in patient-centered mobile solutions. To date, however, there remains a need for an AT app that is appropriate for more advanced adult learning capabilities, is intuitive to use, and is available across mobile platforms.

### Objectives

**Figure 1:**
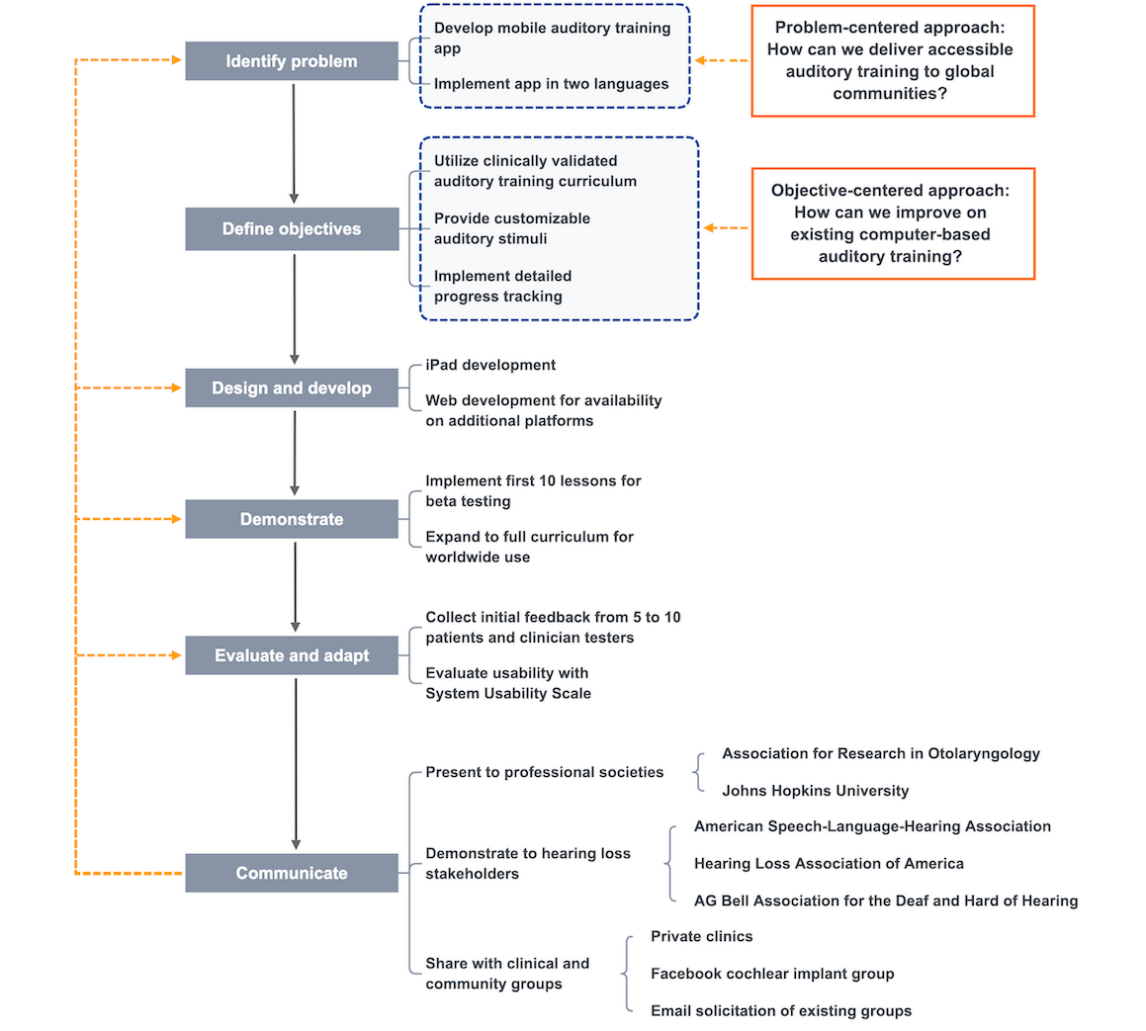
Design Science Research Methodology process model for the Speech Banana app.

To address this need, Speech Banana was developed as a free mobile health (mHealth) app to provide clinically relevant AT on tablet and web platforms. The name alludes to the shape that speech sound frequencies form when visualized on an audiogram. The Design Science Research Methodology (DSRM) [26] was used to produce the Speech Banana app, guiding development with two problem-based and objective-based processes used as research entry points. The problem-based aim was to provide greater worldwide accessibility to AT, and the objective-based aim was to emulate and improve upon clinical techniques (Figure 1). The first aim was addressed by developing the app on two mobile platforms, which provided portability and flexibility, and by implementing the program in English and Korean languages, which demonstrated a successful expansion to international users. The second aim was addressed by using a clinically validated AT program and by implementing features that speech pathologists are often unable to automate during in-person sessions, such as progress tracking and customizing auditory stimuli. These two processes yielded a mental model of the app wherein users could enhance speech comprehension in their own language and at their own pace. This encourages user engagement through feedback from the app and allows clinicians to deliver targeted in-person AT based on users’ at-home progress. The rest of this paper describes the methods for designing, producing, and testing the Speech Banana app; outlines the results of the creation and evaluation processes; and discusses the app’s potential to justify insurance funding for AT and for increased spending toward research on computer-based treatment and telemedicine.

## Methods

Figure 1 summarizes the six components of DSRM and the two nominal processes used as research entry points informing the design.

### Training Model

The training model for Speech Banana aimed to (1) implement a validated AT curriculum, (2) provide a training experience similar to in-person clinic visits, (3) expose users to challenging stimuli that would enhance the generalized application of listening skills learned in the app, and (4) demonstrate that the same training model could be implemented across language systems. These objectives were systematically fulfilled through a review of the existing AT literature and clinical interviews.

To ensure a reliable curriculum, training structures common to in-person AT were identified. AT strategies typically implement a combination of drill exercises that focus on analytic skills and a testing regimen focusing on synthetic skills. Analytic drills include repetition of short words or syllables to enhance sound awareness and discrimination skills, whereas synthetic training involves sentences to cultivate sound identification and comprehension through contextual cues [27,28]. Discussions with clinicians at Johns Hopkins University and Woosong University confirmed that the book Auditory Training for the Deaf [29] closely follows this model, making it an ideal source for the content of Speech Banana. It also isolates narrow frequency windows within each lesson, challenging learners to distinguish between similar phonemes. Although the book was published shortly after World War II, its focus on conversational speech skills remains central to clinical AT today [30]. In addition, the book is available in the public domain via the HathiTrust Digital Library, enabling free distribution of Speech Banana without licensing costs. As an aside, the training plan in Auditory Training for the Deaf was modeled on an earlier book, aptly titled Train Your Hearing [31]. With the book’s curriculum validated, Auditory Training for the Deaf was adopted as the lesson plan for Speech Banana.

To provide an experience similar to in-person training, auditory and visual cues drawn from clinical references were adapted for an app interface. AT clinicians were observed and interviewed at the Listening Center at Johns Hopkins University, to identify methods of implementing the analytic and synthetic regimens. Notable training features included allowing lipreading during exercise drills and hiding the clinician’s mouth during sentence-based tests. Vocal recording and app structure were accordingly designed to reflect these observations, including visual indicators of analytic material (written words) and providing only auditory cues for synthetic material.

To expose users to challenging stimuli that would enhance the generalized application of listening skills, optional background noise was included in the app. Ambient noise was recorded in a café to provide a multitalker babble that competes with the word- and sentence-based stimuli. This reduces the signal-to-noise ratio per user specifications, creating a progressively more difficult listening experience as the background noise level increases.

To show the training model’s applicability across different language systems, a parallel app in Korean was developed. Korean was selected as the second language for Speech Banana because of South Korea’s strong national health care system [32] and widespread adoption of smartphones among aging adults [33]. Lesson material was developed at the Department of Speech Language Therapy and Aural Rehabilitation at Woosong University (Daejeon, South Korea) [34].

### Auditory Stimuli Recording

According to clinical recommendations, spoken auditory stimuli were recorded using clearly enunciated speech to mimic in-person AT and enable easier phoneme discrimination with hearing devices [35-37]. The recording procedures for English and Korean apps are described below.

For the English version, a Zoom H1 Handy Recorder microphone was used with a pop filter to record the words and sentences in Auditory Training for the Deaf [29]. One male and one female speaker with native English fluency completed the recordings in a soundproofed studio at Johns Hopkins University. Speakers maintained a diction pace slightly slower than standard conversational speech to ensure maximum comprehension. Words were read one at a time in a standard American accent, pausing between each word. Sentences were read in a similar fashion, preserving natural intonation, elisions, and stops and enunciating vowels distinctly. Words and sentences were saved as separate files in waveform audio file format, a lossless audio format. Common words used throughout multiple lessons were recorded and saved once and then copied in lessons as required. Audacity audio editing software was used to splice and normalize tracks, ensuring continuity between recording sessions and eliminating cases of clipping or popping.

For the Korean version, a Neumann TLM-170R microphone (Georg Neumann gmBH) and Adobe Premiere Pro CC were used throughout the recording in a studio at Munhwa Broadcasting Corporation (MBC), a Korean television and radio network station. Two male and two female professional broadcasters from MBC recorded sentences for the Korean app. Each pair of male and female speakers performed half of the lessons. After recording, the sound files were transferred to Johns Hopkins University and subsequently normalized using Audacity.

### User Interface

The iPad app was programmed using Xcode, the native language used on the iOS platform. The interface incorporated flat design principles, with few icons, buttons, and navigation links between pages. Contrasting colors and sans serif fonts were used to ensure readability among aging adult users. At the bottom of each page, a Settings button was placed to ensure that features such as speaker sex and background noise level remained accessible throughout the training session. User scores (ie, percentage of correct words and sentences and number of sentences and repetitions) are shown visually as numeric representations. Scores are categorized by lesson, allowing users to compare performance between lessons. To access the app, users must download the software from the Apple App Store onto an iPad.

The web app was programmed in HTML5, JavaScript, and CSS to implement the database and visual elements of the app. The web app was developed using a virtual machine to leverage cloud computing capabilities and allow for app testing on several different operating systems. The web version retained the same color scheme and design principles as the iPad app. User scores (ie, the number of words and sentences correct and number of repetitions) were visualized slightly differently as a bar graph and numeric representation. Scores were categorized by lesson and date of app use, allowing users to compare performance between lessons and over time. To access the web app, users must register with an email address. Upon registration, an identification number is created, and a new user object is created in a MongoDB database to record the quiz performance. Email addresses and passwords were hashed to maintain user security, and no personal health information was stored.

### Communication and Evaluation

To evaluate the performance of the iPad and web apps, 2 mechanisms were employed to spread awareness of Speech Banana and gather user feedback: (1) stakeholder feedback via online forums, academic meetings, and community events and (2) a survey based on the System Usability Scale (SUS), a widely used 10-item assessment tool [38]. Using the first evaluation method, updates to the apps were implemented as user comments were logged. For the second evaluation method, past and present users were invited to complete an SUS survey. Scores were averaged, and performance letter grades were generated through comparison against a normalized scale supported by over 500 studies that used the SUS [39].

## Results

**Figure 2:**
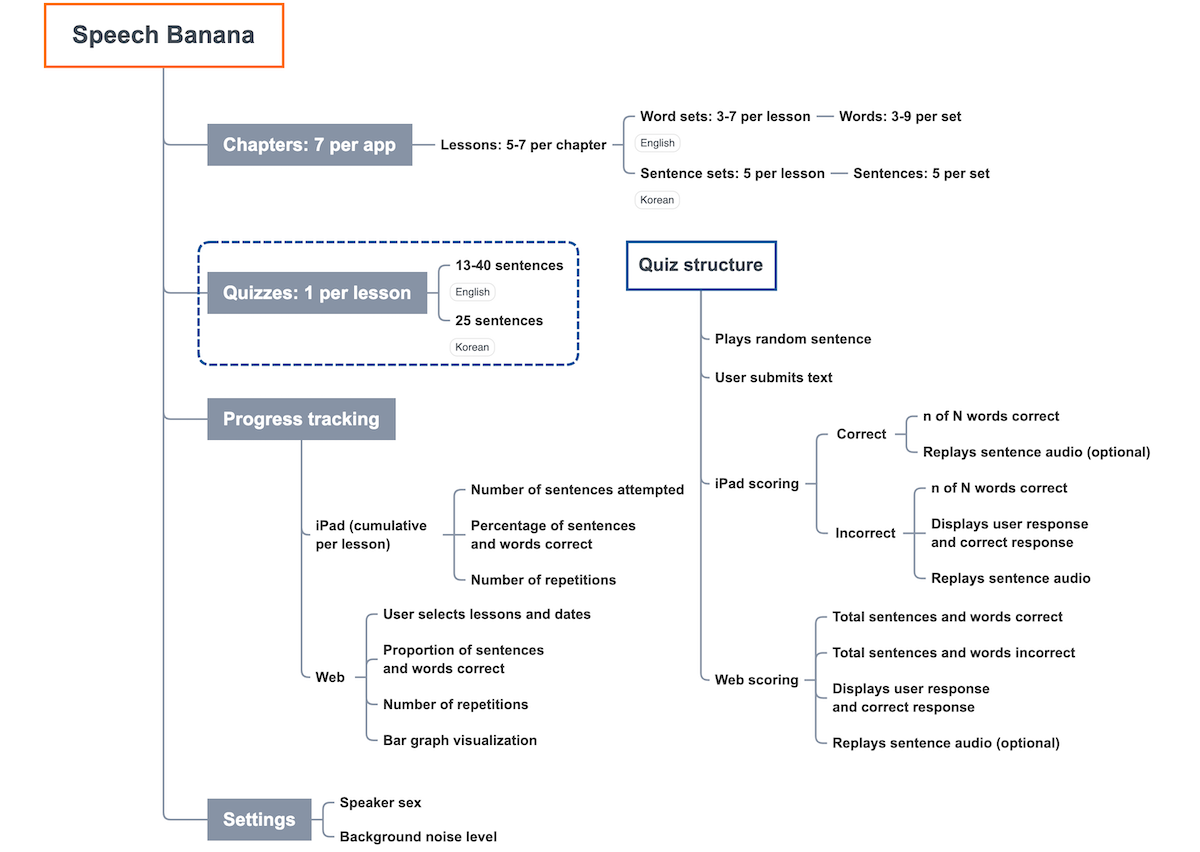
Lesson and quiz structure of the Speech Banana app.

The English and Korean versions of Speech Banana were deployed for the iPad and web platforms. There were more than 3200 downloads of the English iPad app and almost 100 downloads of the Korean app; more than 100 users registered for the web apps. Figure 2 describes the Speech Banana’s structure, function, and usability.

### Training Model

The English app follows Auditory Training for the Deaf [29], with 38 lessons grouped into 7 chapters that cover sounds from low to high frequency (Figure 2). This increases the level of difficulty for users with hearing loss at higher frequencies, a common pattern of hearing loss among aging adults [40]. The first 5 lessons provide an introduction to everyday sounds, including numbers, colors, and conversational phrases. The next 16 lessons focus on vowels: lessons 6-11 address low-frequency vowels, lessons 12-17 incorporate vowels that contain high-frequency components, and lessons 18-21 add diphthongs. The remaining 17 lessons focus on low-to-high frequency consonants, pairing unvoiced, higher frequency consonants with their voiced, lower frequency counterparts. This familiarizes users with more difficult phonemes by associating them with phonemes that are easier to distinguish. All speech phonemes are covered during the course of the lesson plan.

Within each lesson, exercises emphasize analytic training, whereas quizzes incorporate synthetic training. Exercises include visual and auditory stimuli, displaying written words to accompany the sounds of single words and 2-word phrases. Users may repeat these stimuli as needed to practice discrimination and identification skills. After completing a lesson, the app directs users to an interactive quiz that plays auditory-only, sentence-based stimuli (Figure 2, Quizzes). Users may replay sentences before typing their answers into a text box. Throughout the program, lesson word banks increase in size, challenging the user to distinguish larger sets of information. Similarly, quizzes increase in length, providing more sentences and referring to words of earlier lessons to reinforce the material.

The Korean app differs from the English version to accommodate for phonetic and syntactic differences between the two languages and to retain lessons’ progression from low- to high-frequency phonemes [41]. In addition, although the English app contains word and sentence stimuli, the Korean lessons comprise only sentences. This is because existing Korean training and evaluation services use word-based stimuli; however, they lack sentence-based training; therefore, the app was designed to address this training gap. The first 5 lessons comprise brief phrases and sentences, including nouns and verbs, colors, numbers, and common expressions. Following the initial lessons, vowels and consonants are covered in a similar order to the English version. Lessons 6-12 focus on low-frequency vowels, and lessons 13-18 focus on voiced consonants progressing from low to high frequencies. Lessons 19-23 focus on high-frequency vowels, and lessons 24 to 28 focus on unvoiced consonants from low to high frequencies. Lessons 29-33 provide contrasts between vowels and consonants. Lessons 34-35 focus on prosody, such as nursery rhymes, traditional songs, and well-known sports cheers, which are used to encourage engagement with the app. Lessons 36-38 feature advanced auditory discrimination tasks in the form of nonsense sentences. These remove the aid of context clues, helping to develop discrimination acuity and strengthen auditory working memory.

### Auditory Stimuli Recording

Approximately 1170 pairs of words and 1000 pairs of sentences were recorded for the English app. Audio files from lessons 1-10 are freely available to the public on Freesound, an online sound sample repository [42]. These recordings have been used in online games, videos, and educational software. For the Korean app, 950 pairs of sentences were recorded. As the programs include a large number of lossless audio files, the app size is 1.75 GB for the English app and 0.94 GB for the Korean app. This retains high-fidelity playback but occupies considerable storage space on mobile devices.

Both apps showed mild variability in speaker volume or speed. Sporadically, throughout the English language app, the speaker volume varies; meanwhile, in the Korean app, the pair of speakers in the first 19 lessons maintained a slightly quieter tone and faster pace than the second pair. These volume level differences create a more challenging listening experience for new users, and they may be attenuated through additional normalization or rerecording in future app versions.

### User Interface

The apps may be accessed from the Speech Banana web portal [43]. Users may proceed from the portal to the Apple App Store to download the iPad app, or may log into the web app, Google authorization or Naver authorization for the Korean app. Upon entering the app, users encounter the main page that contains motivation for Speech Banana, instructions for use, and links to lessons and quizzes. Curriculum pages have three elements: a header to select speaker sex and choose word sets within a lesson, the sidebar menu to navigate between sections of the app, and the main content space to complete lessons and quizzes.

Lessons contain word or sentence stimuli divided into sets that present materials in manageable learning units. Stimuli are displayed visually in large buttons that users may press to play the associated audio file. At any point in a lesson, users may navigate directly to the corresponding quiz using a gray button on the right side of the page. As users progress through the quiz, scores are updated in real time and are viewable beneath the input bar (web app only) or in the progress section of the app.

During quizzes, users press a button at the center of the page to play a sentence; in response, the app randomly generates a number, searches for the appropriately labeled audio, and plays the file. To ensure that all sentences within the sentence bank are exhaustively selected, numbers are drawn without replacement, and the random function is reset after completing the quiz. Users may play the sentence several times before typing an estimate of what they heard. Input is scored based on syntax and spelling, but punctuation is disregarded. If the written response does not match the auditory stimulus, users’ input and the correct sentences are displayed alongside users’ score, which indicates the number of words typed correctly. The auditory stimulus may then be replayed with the visual stimulus to improve the identification skills.

Background noise and speaker sex may be user-specified throughout the app (Figure 2). When selected, background noise plays continuously at the volume specified by an onscreen slide bar, and the maximum volume is determined by the device’s output capacity. During quizzes, users of the iPad app may select the speaker sex, whereas the web app randomizes speakers using a uniformly distributed random variable.

### Progress Tracking

Scoring on the iPad app is recorded separately for each lesson and displayed on the progress page in a list format (Figure 2). Web app scores are similarly recorded but are visualized with additional tabulation and a dynamic bar chart. Users can display results within a specified period and may filter any of the metrics by lessons or chapters.

### Evaluation and Communication

During development, 2 rounds of feedback were gathered, which helped solidify the user interface and expand to web development. After app publication, communication was primarily conducted among English-speaking audiences and included academic presentations at professional meetings, consultations with clinicians, and demonstrations at community events.

#### Preliminary Feedback

The English version of the iPad app was first developed as an alpha version, consisting of the first 10 lessons and deployed on TestFlight via Apple Store Connect. A total of 10 testers and clinicians were invited to use the app and provide feedback on app navigation, visual esthetic, and auditory levels. After an initial round of feedback, the remaining 28 lessons were implemented.

#### User Feedback

Once the full curriculum was published for iPad use on Apple Store, a continuous stream of feedback was received from the Cochlear Implant Experiences Facebook group, a community page that sees traffic from more than 30,000 members at the time of publication. One salient point of feedback was that people enjoyed the large screen format of a tablet; however, prospective users were unable to access the app because they did not own an iPad. A literature review confirmed this sentiment; although the majority of adults in both advanced and emerging economies use the internet on smartphones, they are much less likely to use computers or tablets [44]. To maximize the accessibility of Speech Banana on any smart device, the team moved forward with web development in English and Korean. The web versions were published in December 2019, and account registration on Google and Naver was added in May 2020. In addition to web development, user comments spurred the creation of online instructions and a frequently asked questions section on the Speech Banana web portal [43]. This helped address common questions regarding app navigation, volume control, and hardware compatibility.

#### SUS Score

Table 1 shows the Speech Banana usability scores from past and present users of the app with letter grades derived from a normalized scale [39].

**Table 1:** Speech Banana usability scores. Letter grades are derived from a normalized scale.

App version	System Usability Scale score, mean (SD)	Grade
English	79.17 (15.39)	B− (Good)
Korean	65.63 (17.99)	D (OK)

The English app received 6 responses to the SUS survey; 4 respondents used the iPad app, 1 used the web app, and 1 used both apps. The Korean app achieved greater SUS participation with 16 scores; 5 from iPad users and 11 from web app users. On the basis of the normalized SUS grades, the English apps achieved good usability, whereas the Korean version performed adequately. The survey results revealed that an initial but brief training session could be implemented to improve user satisfaction and comfort with the apps.

#### Communication

Following the public release of the English iPad app, the app was presented at academic meetings, including the Association for Research in Otolaryngology Annual Meeting, which is the premier conference for research in the auditory and vestibular sciences [45,46]. Talks and posters were additionally presented to clinical stakeholders in the hearing loss community, such as the American Speech-Language and Hearing Association [47]. Finally, updates were shared with clinical and community groups via Johns Hopkins University publications, social media, and electronic mailing lists [48].

## Discussion

### Principal Findings

This study describes the design, development, and assessment of Speech Banana, an mHealth app that responds to a pressing global health need for accessible and effective hearing health care. The app digitizes a validated clinical model that uses word-based exercises to improve users’ familiarity with targeted speech sounds and that delivers sentences-based quizzes to help users generalize their training to conversational listening. Additional features such as tracking enable users to visualize progress with speech comprehension. To make the app available to populations worldwide with multiple languages and access via different electronic devices, Speech Banana was developed in two languages and hosted on two digital platforms. Continuous feedback was used to improve the app, and a pilot usability study rated the mHealth app as adequate to good. These results suggest that the app is successful in providing a usable digital service, and it shows promise as an effective AT option that minimizes the need to pay for or attend clinical rehabilitation sessions. Since its initial worldwide release, Speech Banana has undergone scholarly review, highlighting some of its more successful features and identifying others that would improve the use of the app. Some features have been incorporated into software updates, whereas other features will require future work or technological advancements to fully implement.

With the World Health Organization estimating 466 million people with disabling hearing loss, the lack of accessible AT has become a substantial public health need [49]. Many hearing devices now connect to portable sound amplification products, including smartphones, which have achieved 99% global market penetration [50,51]. This presents an unprecedented opportunity to distribute AT to more people with hearing loss than ever before and to enable users to manage their own hearing health care. The Speech Banana team sought to leverage this opportunity, resulting in an app that is grounded in clinically supported training regimens, implemented in a language with substantially different syntax, and supported across mobile platforms [52].

In designing and executing the Speech Banana app, a 2-pronged design science research approach was chosen, to improve the availability and efficacy of AT worldwide. Traditionally, design science research originates from a single point of entry, which informs subsequent objectives and implementation of the project [26]. From the outset, however, issues were identified both with AT availability and with other AT apps’ delivery and progress tracking methods. The former issue called for the development of a mobile artifact that may be expanded to multiple languages. After initial development and feedback, it became clear that accessibility would not only require an app developed in multiple languages but that the mobile platform itself would significantly impact users’ barriers to entry [44]. With this information, web development was judged to be the most appropriate; therefore, the app could be used on any mobile device with internet access. The latter issue required dedicated attention to the AT curriculum, separate from any technical considerations. Although Auditory Training for the Deaf [29] is out of print, evidence shows that its structure (analytic lessons and synthetic quizzes) is effective for developing speech comprehension skills [27,28]. Furthermore, analytic and synthetic skill building and transition from low- to high-frequency phonemes makes the lesson plan easily adaptable to any language, satisfying both the accessibility and clinical relevance aims. Although the dual-pronged problem statement was nontraditional per established DSRM, it made for a more robust artifact that has strong expansion potential.

Since the release of Speech Banana, additional apps have entered the AT market, and an academic review of these apps has followed [17]. In this review, Olson et al [17] performed a survey of more than 200 mobile apps for AT and deemed 5 iPad apps, including Speech Banana, appropriate for a detailed review. Speech Banana meets the 5 characteristics that were expected of an AT app: (1) it provides feedback through real-time scoring and gives the user an opportunity to repeat the test stimulus, (2) it uses a large training corpus of words and sentences, (3) it trains users on specific phonemes across the speech frequency space, (4) it employs analytic and synthetic processing, and (5) it tracks user performance via a progress report [53]. With these criteria met, Speech Banana is competitive among its peers in the AT app space.

### Limitations and Future Work

Although Speech Banana addresses the original aims, it should be noted that there are several limitations that merit addressing in future development of the app, or that may require technological advances. First, using lossless files for auditory stimuli leads to a large total app size, which consumes a substantial portion of the available memory space on current iPads. These files are necessary to ensure clear audio for users learning to hear; however, it may deter some users from downloading the app. As mobile storage space continues to increase, space limitations may become less of a concern. For users who do not own an iPad, use of the web app may be infeasible with current internet connectivity; however, as internet access becomes increasingly ubiquitous, this limitation will diminish, enabling easier distribution of Speech Banana and mobile AT more broadly. Second, although Speech Banana’s stimuli are of high quality, the auditory output may be limited by the capabilities of iPad or smartphone speakers. This may be mitigated by using headphones, direct audio input, or Bluetooth connectivity. Third, users mentioned volume inconsistency across the sound files. This may be reduced by reprocessing the raw audio files or through further normalization to account for the recording of batch effects. Fourth, older users found it difficult to type responses in the quiz windows. This can be significantly alleviated by using a stylus pencil that works with the newer iPad models.

Apart from basic functionality limitations, feedback has suggested several feature additions that could improve user experience, including phonemic progress tracking, adaptive quizzes, and additional languages. Phonemic progress tracking would significantly advance the app’s integration with periodic in-person AT. Word- and sentence-based achievement used in the current version of the app does not inform users of frequency or timing cues that require additional training. If the app tracked phoneme comprehension, Speech Banana could better aid targeted clinical interventions or guide the calibration of hearing devices. One potential method could employ cloud computing to classify and interpret trends in phoneme comprehension errors. In addition to improving speech comprehension, phonemic tracking could be used to train the vocalization of challenging phonemes, enhancing speech production. Adaptive quizzes can improve user engagement and prevent frustration by adjusting the difficulty based on previous performance. This may elongate the session length, increasing the potential efficacy of the app. Language additions have been requested for several languages, including British English, French, German, Turkish, Arabic, Spanish, Hindi, Tamil, and Sinhalese. These would enhance global accessibility, but developing the app is time consuming, particularly with a small team. Implementation may be accelerated by using publicly available speech corpora such as the British English Speech Corpus, which is frequently used in speech recognition research. To ensure phonemic consistency across languages, speech and language clinicians should be invited to collaborate on curriculum development.

Finally, although Speech Banana uses a curriculum that conforms to clinical AT methods, the app itself has not been validated for efficacy, skill generalization, computer-based AT compliance, and training durability [1,54-56]. To address this, a randomized clinical trial should be conducted to evaluate the use of the app on different mobile devices, comparing efficacy among patients with and without access to supplemental in-person AT [52,57]. As clinical interest in telemedicine expands, this could serve as evidence to justify increased private and public insurance funding toward AT availability and toward the development of AT technologies.

As the global aging population grows, the incidence of hearing loss continues to rise, requiring that options for AT are made accessible to mitigate or forestall cognitive decline [58]. Isolation because of the recent COVID-19 pandemic has demonstrated the necessity of telemedicine advancements and has provided evidence that remote health care can be effective with careful design and implementation. Continued use and evaluation of the Speech Banana app will help us understand the efficacy of computer-based AT and will aid the development of coordinated care that integrates clinical time with home practice. Through the current app and future extensions, Speech Banana may be an effective tool to broaden access to hearing health care worldwide.

## Abbreviations

AT: auditory training
DSRM: Design Science Research Methodology
MBC: Munhwa Broadcasting Corporation
mHealth: mobile health
SUS: System Usability Scale
